# ﻿Taxonomic review of *Gallardonerisnonatoi* (Ramos, 1976) comb. nov. (Annelida, Lumbrineridae), and description of a new species of *Lumbrineris* from the Gulf of Mexico

**DOI:** 10.3897/zookeys.1114.79492

**Published:** 2022-07-25

**Authors:** Daniel Martin, Jordi Estefa, João Gil

**Affiliations:** 1 Centre d’Estudis Avançats de Blanes (CEAB-CSIC), carrer d’accés a la Cala Sant Francesc 14, ES17300 Blanes, Catalunya, Spain Centre d’Estudis Avançats de Blanes Blanes Spain; 2 Department of Organismal Biology, Evolutionary Biology Centre, Uppsala University, P. O. Box 256, SE-751 05 Upsala, Sweden Uppsala University Upsala Sweden; 3 Centre of Marine Sciences (CCMAR), University of Algarve, Campus de Gambelas, 8005-139 Faro, Portugal University of Algarve Faro Portugal

**Keywords:** *Gallardonerisiberica* syn. nov. misidentification, morphometry, new combination, Polychaeta, South European Atlanto-Mediterranean, synonymy

## Abstract

The small Lumbrineridae*Gallardonerisiberica* Martins, Carrera-Parra, Quintino & Rodrigues, 2012 was first described as new to science based on specimens from Portuguese waters. Then, it was successively reported from several south European areas, including Spain, Italy, Greece, and Cyprus. Here evidence is presented that *G.iberica* should be placed in synonymy with *Lumbrinerisnonatoi* Ramos, 1976, originally described from NW Mediterranean waters, a species that fits with the diagnosis of *Gallardoneris*. Based on specimens from the French coasts of the NW Mediterranean, this paper (1) redescribes the species using the new combination *Gallardonerisnonatoi* (Ramos, 1976) and (2) provides a morphometric analysis of its main morphological characters. The lack of recent reports of *G.nonatoi***comb. nov.** in Mediterranean waters is presumably due to the recent redescription of the species as *L.nonatoi* based on specimens from the Gulf of Mexico. However, these specimens belong to *Lumbrineris*, as currently defined. By assessing their morphological differences, it is concluded that the specimens from the Gulf of Mexico represent a different and new species, namely *Lumbrinerisjan***sp. nov.** Also discussed is the possible assignation of *Lumbrinerislongipodiata* Cantone, 1990, a poorly known species seldom recorded since its original description from the Gulf of Catania (Mediterranean Sea) to *Gallardoneris*, as well as on whether it is a valid species or may be an additional junior synonym of *G.nonatoi***comb. nov.**

## ﻿Introduction

The family Lumbrineridae Schmarda, 1861 (Annelida) has received renewed attention during the last two decades, mostly thanks to the efforts of Luis Fernando Carrera-Parra and collaborators, who have reviewed totally or partially some of its genera, such as *Cenogenus* Chamberlin, 1919 (Carrera-Parra 2001b), *Kuwaita* Mohammad, 1973 (Carrera-Parra and Orensanz 2002), *Lumbricalus* Frame, 1992 (Carrera-Parra 2004), and *Lumbrineris* de Blainville, 1828 (Carrera-Parra 2006b), occasionally with the support of phylogenetic analyses (Carrera-Parra 2005, 2006a). More recently, the first partial revision of Lumbrineridae based on molecular data confirmed the monophyly of *Abyssoninoe* Orensanz, 1990, *Augeneria* Monro, 1930, *Gallardoneris* Carrera-Parra, 2006, *Lumbrineriopsis* Orensanz, 1973 and *Ninoe* Kinberg, 1865, while *Helmutneris* Carrera-Parra, 2006, *Hilbigneris* Carrera-Parra, 2006 and *Lumbrinerides* Orensanz, 1973 were recovered as independent genera, and *Lumbrineris* and *Scoletoma* de Blainville, 1828 were considered polyphyletic (Borisova and Budaeva 2022).

The studies by Carrera-Parra and co-authors also triggered numerous collaborations dealing with local faunas, some of them covering the waters of the Iberian Peninsula (Martins et al. 2012; Arias and Carrera-Parra 2014; García Gómez et al. 2016) or the Mediterranean Sea (Carrera-Parra et al. 2011; Katsiaras et al. 2018). These, combined with other studies (Gil 2011; Bertasi et al. 2014; D’Alessandro et al. 2014; D’Alessandro et al. 2016; Kurt-Sahin et al. 2016; Aguirrezabalaga and Arias 2018; Langeneck et al. 2020), reported ~ 35 lumbrinerid species for the Iberian Atlanto-Mediterranean waters and ~ 30 for the whole Mediterranean Sea, belonging to the genera *Abyssoninoe*, *Augeneria*, *Cenogenus*, *Eranno* Kinberg, 1865, *Gallardoneris*, *Helmutneris*, *Hilbigneris*, *Kuwaita*, *Lumbricalus*, *Lumbrinerides*, *Lumbrineriopsis*, *Lumbrineris*, *Ninoe*, and *Scoletoma*.

Among them, *Gallardoneris* was first described to include two Indo-Pacific species, *G.shiinoi* (Gallardo, 1968), from Vietnam, and *G.thailandensis* Carrera-Parra, 2006, from Thailand (Carrera-Parra 2006a). More recently, *Gallardonerisiberica* Martins, Carrera-Parra, Quintino & Rodrigues, 2012 was described from Portuguese waters (Iberian Peninsula, NE Atlantic) as being the third species of the genus (Martins et al. 2012). Shortly after it was reported from Italy (Bertasi et al. 2014; D’Alessandro et al. 2016), the Spanish Atlanto-Mediterranean coasts of the Iberian Peninsula (García Gómez et al. 2016), Greece and Cyprus (Katsiaras et al. 2018).

Through the analysis of numerous specimens from multiple surveys in the western Mediterranean Sea (but mainly in Spanish and French waters), we realised that those usually identified as *Lumbrinerisnonatoi* Ramos, 1976, a species originally described from the western Mediterranean, fitted perfectly with the description of the newly reported *G.iberica*. *Lumbrinerisnonatoi* had been commonly recorded in that sector from shallow shelf soft sediments for more than 30 years, and had also been widely reported across the whole Mediterranean Sea from areas such as the Gulf of Lions (Labrune et al. 2007), Italian waters (Castelli et al. 1995), Adriatic Sea (Mikac 2015), Crete (Papadopoulou et al. 1994), Cyprus (Çinar 2005), or Turkey (Çinar and Dagli 2013). However, it stopped being reported after the description of *G.iberica* by Martins et al. (2012).

As part of his taxonomic works on lumbrinerids, Carrera-Parra (2001a, 2006b) provided a redescription of *L.nonatoi*. In the absence of more suitable material, this redescription was based on specimens from the Gulf of Mexico, while the original description corresponded to specimens from the Catalan Sea (western Mediterranean) (Ramos 1976). As a result, *L.nonatoi* was retained in *Lumbrineris* (Carrera-Parra 2001a, 2006b) and, as such, joined other species of the genus in recent identification keys for the Mediterranean and southwest European Atlantic waters (Martins et al. 2012; D’Alessandro et al. 2016; García Gómez et al. 2016; Aguirrezabalaga and Arias 2018). Considering the reported characters, however, it is now clear that the redescription and the original description refer to different species, with the Mexican specimens belonging to *Lumbrineris*, as currently defined (Carrera-Parra 2006a).

Four consequences derive from this misidentification. First, the recently described *G.iberica* should be considered a junior synonym of *L.nonatoi* sensu Ramos (1976) and, thus, all successive citations of the former from European Atlanto-Mediterranean waters should presumably be assigned to the latter (e.g., Martins et al. 2012; Bertasi et al. 2014; D’Alessandro et al. 2016; García Gómez et al. 2016; Aguirrezabalaga and Arias 2018; Katsiaras et al. 2018). Second, according to the generic diagnostic characters described by Carrera-Parra (2006b), the European *L.nonatoi* clearly represents a species of *Gallardoneris*. Third, no European lumbrinerid can be correctly identified as “*Lumbrinerisnonatoi*” using the most recent literature for the region, which either refers to the misidentified specimens from the Gulf of Mexico (not present in European waters), or to a chimera composed of characters from the specimens of the Gulf of Mexico mixed with features from the original description of *L.nonatoi*. Fourth, the specimens from the Gulf of Mexico attributed to “*Lumbrinerisnonatoi*” by Carrera-Parra (2001a; 2006b) belong instead to a new species.

## ﻿Materials and methods

Numerous specimens of *Lumbrinerisnonatoi* sensu Ramos (1976) collected from the Mediterranean coasts of Spain and France, were studied and used to confirm the accurate original description by Ramos (1976). A selection of 40 entire specimens from the French Mediterranean coasts were used for morphometric measurements of body length and width, prostomium and peristomium length and width, length and width at chaetigers 10 and 15, total number of chaetigers, and number of chaetigers with composite hooks and bilimbate chaetae. The relationships between the quantitative characters measured were estimated by linear regression and plotted using the statistical software XLSTAT 2015 (Addinsoft).

Denotation of maxillary elements follows Carrera-Parra (2006a) and Martins et al. (2012) and, thus, **MI–MIV** corresponds to maxillae I to IV. Other abbreviations in the text are: **CMHH** = composite hooded hooks; **D1–D2** = first and second teeth pair, according to Ramos (1976); **L** = length; **N** = number of examined specimens; **p** = significance level (probability); **post**: postchaetal lobe; **pre**: prechaetal lobe; **R^2^** = Square Pearson coefficient; **SMHH** = simple hooded hooks; **W** = width.

Representative specimens of *Lumbrinerisnonatoi* sensu Ramos (1976) are deposited in the collections of the Centre for Advanced Studies of Blanes (**CEAB**) of the High Council for Scientific Research (**CSIC**, Spain). Type and non-type materials of *G.iberica* were loaned from the Biological Research Collection of Marine Invertebrates (**COBI**) of the Biology Department of the University of Aveiro (Portugal).

The Mexican specimens of *Lumbrinerisnonatoi* sensu Carrera-Parra (2001a; 2006b) are here described as a new species. The description is provided in accordance with article 72.4.2 of the International Code of Zoological Nomenclature (International Commission on Zoological Nomenclature 1999), concerning new nominal species-group taxa being based on a published misidentification by an earlier author. While we did not follow recommendation 73B of the Code (International Commission on Zoological Nomenclature 1999) in that “An author should designate as holotype a specimen actually studied by him or her, not a specimen known to the author only from descriptions or illustrations in the literature”, the materials were perfectly and accurately described by Carrera-Parra (2001a, 2006b). Therefore, no additional observations of new materials or samples were found to be necessary. The holotype and paratypes of the new species were selected with the assistance of Dr Carrera-Parra among the specimens deposited in the Reference Collection of Benthos of ECOSUR-Chetumal, Mexico (**ECOSUR**), in the Colección de Poliquetos del Instituto de Ciencias del Mar y Limnología, UNAM, Mexico (**CP-ICML-UNAM**), and in the National Museum of Natural History, Smithsonian Institution, Washington D.C., USA (**USNM**). Additional information concerning the types was retrieved from Uebelacker (1984), Carrera-Parra (2001a, 2006b), and GBIF Secretariat (2021).

The electronic version of this article in portable document format (PDF) will represent a published work according to the ICZN (International Commission on Zoological Nomenclature 1999), and hence the new names contained in the electronic version are effectively published under that Code from the electronic edition alone.

## ﻿Taxonomic account

**Family Lumbrineridae Schmarda, 1861**

### ﻿Genus Gallardoneris

Taxon classificationAnimaliaEunicidaLumbrineridae

Carrera-Parra, 2006

A3BF19EA-00B3-5A4B-8953-07CFC911BAFF

#### Type species.

*Lumbrinerisshinoii* Gallardo, 1968

#### Diagnosis.

Emended from Carrera-Parra (2006a). Lumbrinerids lacking antennae and branchiae, with (?) or without anal cirri. Notopodia slightly developed. Maxillary apparatus with four pairs of maxillae; MI forceps-like lacking internal accessory teeth, wide base, lacking attachment lamella; MII as long as MI, with ligament, lacking attachment lamella and connecting plates with MI; MIII completely pigmented, with a narrow attachment lamella along 1/4 of its posterior lateral edge; MIV with a whitish central area and narrow attachment lamella; carriers joined to entire base of MI; mandibles totally fused. Simple and composite multidentate hooded hooks present; limbate simple multidentate hooded hooks absent.

#### Remarks.

*Gallardoneris* was originally diagnosed as having anal cirri, but they were not described or pictured for any of the species originally included in the genus (Gallardo 1968; Carrera-Parra 2006a), while they are absent in *G.nonatoi* comb. nov. Therefore, it is not possible to state whether the genus can be exclusively characterized by lacking anal cirri, as occurs in the European specimens. Consequently, the dual possibility (having or lacking anal cirri) is kept in the diagnosis, though the genus probably lacks them.

### ﻿ Gallardoneris nonatoi

Taxon classificationAnimaliaEunicidaLumbrineridae

(Ramos, 1976) comb. nov.

EC8A3ED3-6ED2-5D06-92A3-B3941328F0CA

**Figure 1.**
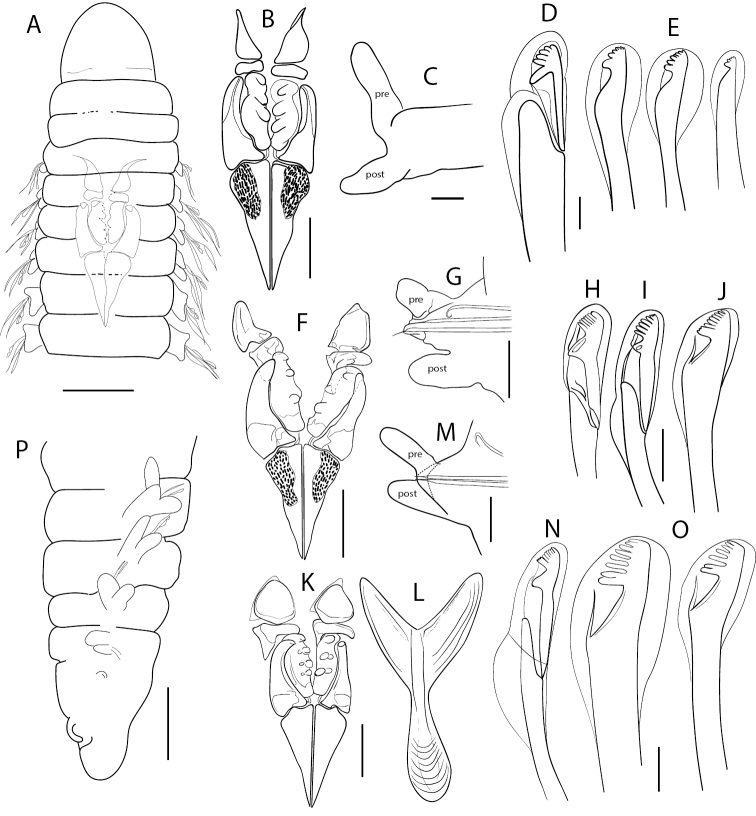
*Gallardonerisnonatoi* (Ramos, 1976) comb. nov. **A** anterior end **B, F, K** maxillary apparatus **C, G, M** parapodia from midbody, chaetiger 88 and chaetiger 65, respectively; pre: prechaetal lobe; post: postchaetal lobe **D, H, I, N** composite hooded hooks **E, J, O** simple hooded hooks **L** mandibles **P** posterior end **A–E, P** redrawn from Ramos (1976)**F–J** redrawn from Martins et al. (2012)**K–O** redrawn from Bertasi et al. (2014). Scale bars: 10 µm (**D, E, N, O**); 12 µm (**H–J**); 25 µm (**G**); 50 µm (**A, M**); 100 µm (**B, C, P**); 200 µm (**F, K, L**).

**Figure 2.**
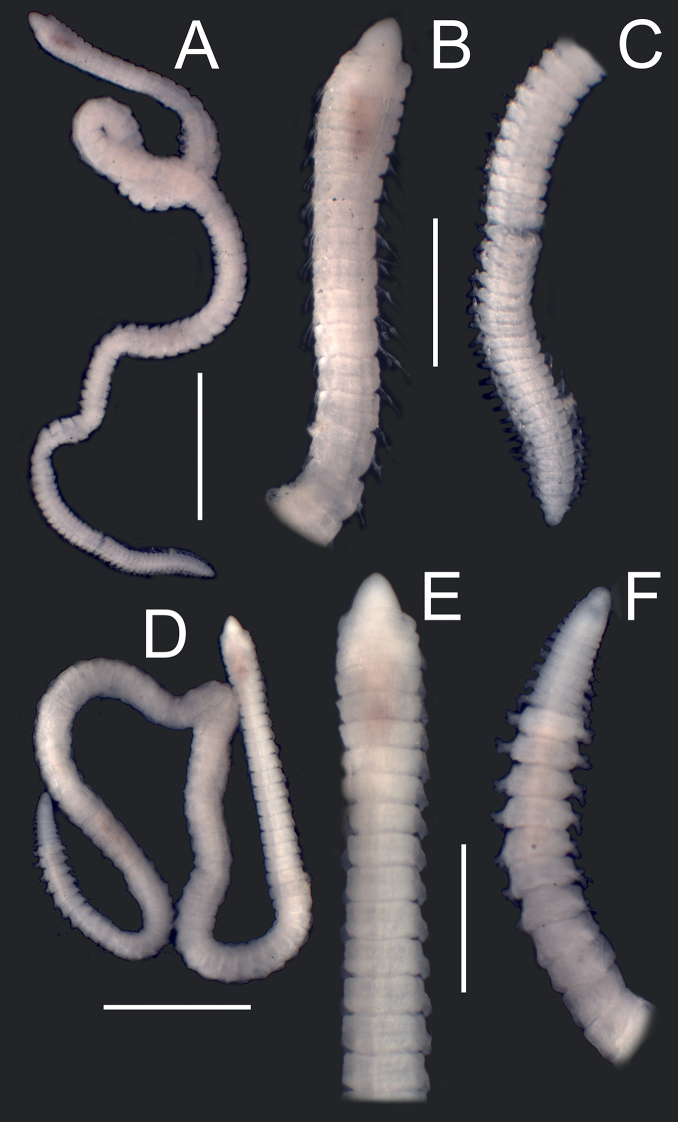
*Gallardonerisnonatoi* (Ramos, 1976) comb. nov. Stereomicroscope photos of entire specimens. **A, D** whole body, dorsal view **B, E** anterior end, dorsal view **C, F** posterior end, dorsal view (**C** normal; **F** regenerating last segments). Scale bars: 1.6 mm (**A, D**), 0.6 mm (**B, C, E, F**).

**Figure 3.**
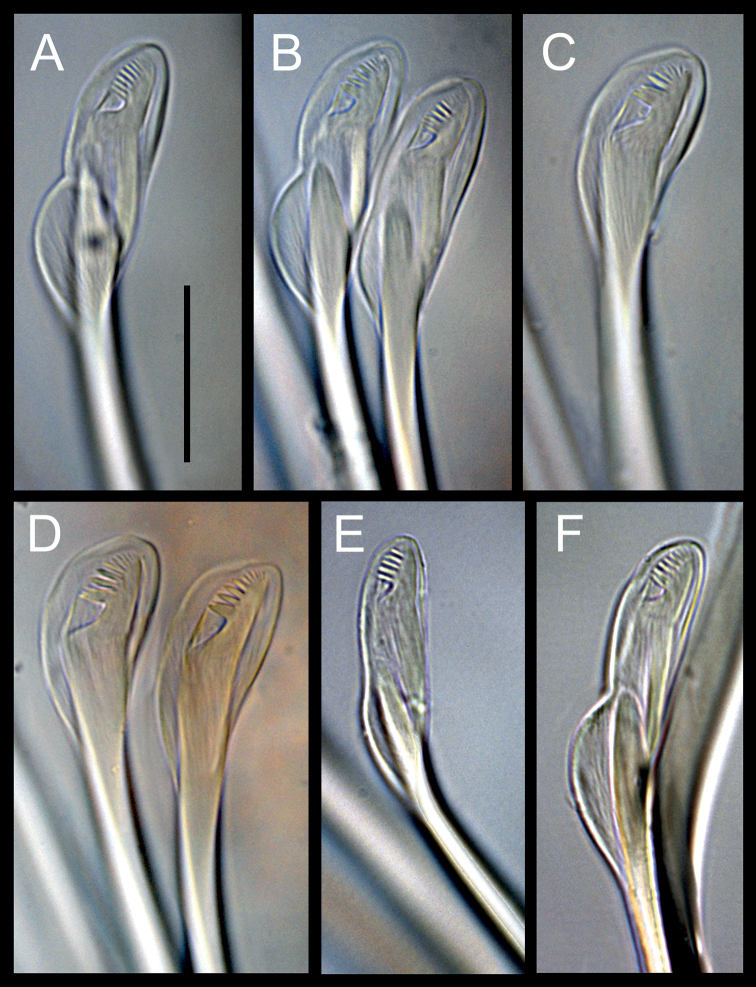
*Gallardonerisnonatoi* (Ramos, 1976) comb. nov. Binocular microscope photos **A, B, E, F** composite hooded hooks **C, D** simple hooded hooks **A–D** from the Gulf of Fos (South France, Mediterranean Sea) **E** from the Portuguese continental shelf (Atlantic Ocean) **F** from Barcelona (Catalunya, Mediterranean Sea). Scale bar: 20 µm.

**Figure 4.**
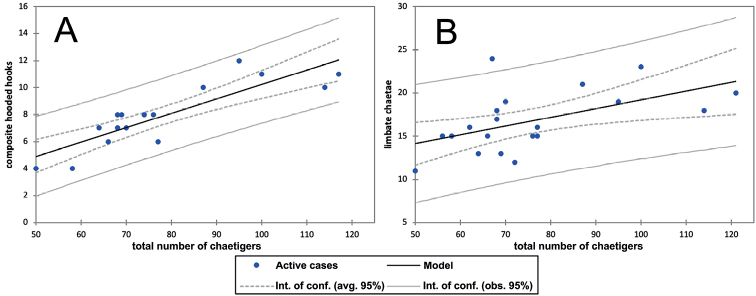
*Gallardonerisnonatoi* (Ramos, 1976) comb. nov. Morphometric relationships showing the regression model and the intervals of confidence (95%) over the average (avg.) and observed (obs.) values. Number of chaetigers with composite hooded hooks (**A**) and bilimbate chaetae (**B**) vs. total number of chaetigers.

**Figure 5.**
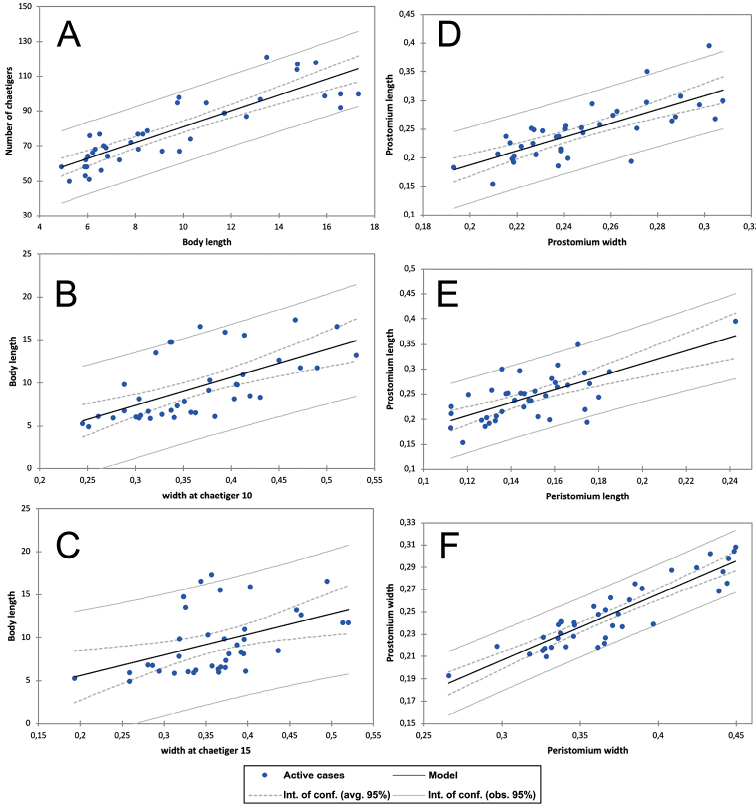
*Gallardonerisnonatoi* (Ramos, 1976) comb. nov. Morphometric relationships showing the regression model and the intervals of confidence (95%) over the average (avg.) and observed (obs.) values. **A** number of chaetigers vs. total body length (mm) **B** body length (mm) vs. body width at chaetiger 10 (mm) **C** body length (mm) vs. body width at chaetiger 15 (mm) **D** prostomium length (mm) vs. width (mm) **E** prostomium length (mm) vs. peristomium length (mm) **F** prostomium width (mm) vs. peristomium width (mm).

Figs 1 , 2 , 3 , 4 , 5

Lumbrineris nonatoi Ramos, 1976: 124–127, figs 19–21 – Campoy (1982): 605–606; Papadopoulou et al. (1994): 263; Gil (2011): 398.
Gallardoneris iberica Martins, Carrera-Parra, Quintino & Rodrigues, 2012: 6–10, fig. 2 – Bertasi et al. (2014): 3–5, figs 2–4; D’Alessandro et al. (2016): 236–237, fig. 4; García Gómez et al. (2016): 1430–1432, fig. 3A; Katsiaras et al. (2018): 1611–1614, figs 2–3. [Syn. nov].

#### Material examined.

Western Mediterranean Sea. CEAB AP 986A, 3 specimens, Gulf of Fos, France, approx. 43.41°N, 04.93°E, 2008, coll. Creocean; CEAB AP 986B, 2 specimens, Gulf of Fos, France, approx. 43.41°N, 04.93°E, 2009, coll. Creocean; CEAB AP 986C, 30 specimens, Quai de Séte, France, approx. 43.40°N, 3.72°E, 2009, coll. Creocean; CEAB AP 986D, 5 specimens, Port la Nouvelle, France, approx. 43.01°N, 03.08°E, 2010, coll. Creocean. All specimens fixed with a 10% formalin/sea water solution, stained with Rose Bengal and preserved in 70% ethanol.

#### Additional material.

*Gallardonerisiberica*: 1 ***paratype*** (DBUA0001315.01), preserved in 96% ethanol, NW continental shelf of Portugal, Atlantic Ocean, 39°48.584'N, 09°13.773'W, 100.5 m, fine sand, MeshAtlantic St. 3B, coll. R. Martins, June 2010; 3 non-type specimens (DBUA0001457.01), preserved in 70% ethanol, off Barcelona, Catalonia, Spain, western Mediterranean Sea, 41°23'27.14"N, 02°12'56.58"E, 21 m, muddy sand, coll. S. García Gómez, October 2013.

#### Description.

Prostomium conical, variable, short, almost equally longer than wide, lacking antennae and eyes; peristomium shorter and clearly wider than prostomium, with two rings of similar size; junction of prostomium with peristomium normally forming an open angle (Figs 1A, 2A, B, D, E). Maxillary apparatus with four pairs of maxillae; maxillary carriers as long as maxillae I (MI), almost triangular, joined along base of MI; MI forceps-like with wide recurved base, without attachment lamella; MII stout, as long as MI, with ligament, with three teeth, without attachment lamella; MIII edentate; MIV edentate plate, with lighter central area (Fig. 1B). Mandible completely fused, Y-shaped. Prechaetal lobe inconspicuous in most anterior chaetigers, becoming ovoid and longer along body until being clearly digitiform and longer than postchaetal lobe in most posterior chaetigers; postchaetal lobe auricular in anterior segments, becoming digitiform from ca. chaetiger 20 (Fig. 1C). Bilimbate capillary chaetae from chaetiger 1 to 7–12 dorsally and from 1 to 24–30 ventrally, with limb typically forming a clearly asymmetrical basal swelling, tapering swiftly to a slender and long tip curving smoothly but clearly on same side of basal swelling. Composite multidentate hooded hooks up to chaetiger 9 (ranging from 4–12, Fig. 4A), with very short blades, 6–8 teeth either all similar or with proximal tooth bigger (due to partial fusion of several smaller teeth) and a long hood, and a characteristic second swelling at shaft end, at shaft/blade joint level (Figs 1D, 3A, B, E, F); replaced from chaetiger 10 by simple multidentate hooded hooks, with seven or eight teeth similar in size, proximal one larger due to partial fusion of several smaller teeth (Figs 1E, 3C, D). Up to four aciculae, yellow, aristate, reducing to one or two in posterior parapodia. Pygidium conical, lacking anal cirri; anus dorsal (Figs 1P, 2C, F).

#### Type locality.

Gulf of Roses (= Bay of Roses), Catalan Sea (northwestern Mediterranean, 42°15'6"N, 3°9'6"E), 10 m depth, sandy mud (Ramos 1976).

#### Known distribution.

Western and eastern Mediterranean Sea, Adriatic Sea, Aegean Sea, Levantine Sea; Atlantic coasts of the Iberian Peninsula; on sandy and muddy bottoms; 4–180 m depth.

#### Morphometry.

*Gallardonerisnonatoi* comb. nov. is a relatively small species, and the studied population ranged from slightly < 5 mm to ~ 17 mm long. This character showed the largest size-related variability (130%), followed by the number of chaetigers with composite hooded hooks (100%) (Table 1, Fig. 4A). The lowest variability occurred in prostomium and peristomium width (slightly < 40%), followed by the widths at chaetigers 10 and 15 (between 50% and 60%) (Table 1, Fig. 5B, C, F). However, some characters proved to be significantly size-related (Table 1, Figs 4A, B, 5A–F). The best correlation occurred between prostomium and peristomium width, followed by the number of chaetigers vs. total body length and number of chaetigers with composite hooded hooks vs. total number of chaetigers (Table 1, Figs 4A, 5A, F). The weakest correlation occurred between number of chaetigers with bilimbate chaetae and total number of chaetigers, followed by body width at chaetiger 10 and total body length (Table 1, Figs 4B, 5B). Moreover, this relationship was much weaker than that between body width at chaetiger 15 and total body length (Table 1, Fig. 5B, C), indicating that the latter better represented the total size in case on finding fragmented worms.

**Table 1.** Measurements and relationships (linear regressions) for the main characters of *Gallardonerisnonatoi* comb. nov. Abbreviations: *N* = number of examined specimens; p = significance level (probability); R^2^ = Square Pearson coefficient.

Morphometric characters	Average	Minimum	Maximum	*N*
Total body length (mm)	9.50	4.92	17.31	40
Length at chaetiger 10 (mm)	1.48	1.20	1.94	40
Length at chaetiger 15 (mm)	2.18	1.64	2.90	40
Width at chaetiger 10 (mm)	0.36	0.24	0.53	40
Width at chaetiger 15 (mm)	0.36	0.19	0.52	40
Total number of chaetigers	78.44	50	121	39
Number of chaetigers with composite hooks	7.94	4	12	16
Number of chaetigers with bilimbate chaetae	16.75	11	24	20
Prostomium length (mm)	0.24	0.15	0.35	40
Prostomium width (mm)	0.25	0.19	0.31	40
Peristomium length (mm)	0.15	0.11	0.24	40
Peristomium width (mm)	0.37	0.27	0.45	40
Linear regressions			R^2^	p
Number of chaetigers = 35.782+4.532*Body length			0.749	< 0.0001
Total body length = -2.399+32.663*Width at 10			0.187	0.005
Total body length = 0.843+23.868*Width at 15			0.383	< 0.0001
Prostomium length = -0.055+1.213*Prostomium width			0.576	< 0.0001
Prostomium length = 0.052+1.295*Peristomium length			0.457	< 0.0001
Prostomium width = 0.028+0.595*Peristomium width			0.814	< 0.0001
Chaetigers with bilimbate chaetae = 9.092+0.101*Total chaetigers			0.291	0.014
Chaetigers with composite hooks = -0.392+0.106*Total chaetigers			0.740	< 0.0001

#### Remarks.

All Mediterranean specimens studied fully agree with the original description of the species by Ramos (1976), with minor morphometric and morphological variation, both in composite and simple hooks and parapodial characteristics. Some of them may result from the size-related morphometric relationships described above, but also from differences in observation procedures and drawing interpretations (Tables 1, 2, Figs 1B–O, 3A–F, 4A, B, 5A–F).

**Table 2.** Main morphological characteristics of *Gallardonerisnonatoi* comb. nov. compared to *Lumbrinerisjan* sp. nov. Abbreviations: CMHH = composite hooded hooks; L = length; SMHH = simple hooded hooks; W = width.

	*Gallardonerinonatoi* (Ramos, 1976) comb. nov.							*Lumbrinerisjan* sp. nov.
	(Ramos 1976)	Martins et al. (2012)	Bertasi et al. (2014)	García Gómez et al. (2016)	D’Alessandro et al. (2016)	Katsiaras et al. (2018)	This paper	Carrera-Parra (2001a, 2006b); this paper
Distribution	Catalan Sea	Portugal	Adriatic Sea	Catalan Sea	Tyrrhenian Sea	Eastern Mediterranean	French NW Mediterranean	Gulf of Mexico
Prostomium	conical, variable	conical, variable	subconical to ovoid	conical, variable	conical	conical, variable	conical, variable	globular, as long as wide
L10 (mm)	0.6–1.32	1.2–1.3	0.32–0.57	–	–	0.27 – 2.05	1.20–1.93	1.3 – 2.7
W10 (mm)	0.4	0.2–0.5	1.45–2.02	0.4	–	0.16 – 0.54	0.24–-0.53	0.3 – 0.9
Segments (max. number)	87	101	76	78	–	82	121	
Mandibles	fused	fused	fused	fused	fused	fused	fused	slightly divided
Number of maxillae pairs	4	4	4	4	4	4	4	5
MII (left right)	3+3	3(4)+3	3(4)+3	3+3	3+3	3+3	3+3	3+3
MIII	edentate	edentate	edentate	edentate	edentate	edentate	edentate	unidentate
MIV	edentate, white central area	edentate, white central area	edentate, white central area	edentate, white central area	edentate, white central area	edentate, white central area	edentate, white central area	unidentate
MV	absent	absent	absent	absent	absent	absent	absent	present
CMHH								
Blade size*	short	short	short	short	short	short	short	short
Teeth number	7–8	7	7	7	7	up to 7	6–8	up to 5
Teeth size	similar	proximal bigger	proximal bigger, partially fused with next one	proximal bigger	proximal bigger	proximal bigger	proximal bigger	all similar
Hood	long, with second swelling	long, with second swelling	long, with second swelling	long, with second swelling	long, with second swelling	–	long, with second swelling	long, with second swelling
SMHH								
First chaetiger	10	7–10	7–10	8	–	7–10	5–13	7–20
Teeth number	9	7	7	7	–	up to 7	7–8	up to 6
Teeth size	proximal bigger	proximal bigger	proximal bigger	proximal bigger	proximal bigger	proximal bigger	proximal bigger	proximal bigger
SMHH hood	long	long	long	long	long	–	long	short
Preacicular size	twice	equal	twice	–	–	twice	twice	equal
Bilimbate chaetae								
Last ventral (chaetiger)	–	7–13	8–12	–	–	7–9	7–12	19
Last dorsal (chaetiger)	–	25–35	18–19	–	–	17–22	24–30	–
Prechaetal lobe								
Anterior (shape)	ovoid	inconspicuous	inconspicuous	–	inconspicuous	ovoid	inconspicuous	elongated
Posterior (size)	long	small/similar/long	small/similar/long	small/similar/long	small/long	longer	small/similar/long	longer
Posterior (shape)	–	ovoid then digitiform	ovoid then digitiform	–	digitiform	ovoid then digitiform	ovoid then digitiform	digitiform
Postchaetal lobe								
Anterior (shape)	cylindrical	auricular	auricular	auricular	–	auricular	auricular	digitiform
Posterior (shape)	digitiform	digitiform	digitiform	digitiform	digitiform	digitiform	digitiform	digitiform
Aciculae								
Dorsal	curved	?	curved	–	–	?	curved	several, curved
Ventral (colour)	yellow	yellow	yellow	–	–	yellow	yellow	yellow
Ventral (maximum number)	1?	2	4	–	3	4	2	up to 3
Anal cirri	absent	absent	absent	absent	absent	absent	absent	two

*Gallardonerisnonatoi* comb. nov. characteristically lacks anal cirri, a feature seldom observed among lumbrinerids. They are also absent in members of *Lumbrinerides* and *Lumbrineriopsis*, two genera that also coincide in lacking MV. However, these two genera form a cohesive group sharing other characters that are absent in *Gallardoneris*, including having bidentate simple hooded hooks, lacking composite hooks, or having completely pigmented MIV (Carrera-Parra 2006a). The lack of anal cirri in *G.nonatoi* comb. nov. in relation to *Lumbrinerides*/*Lumbrineriopsis* would thus be due to homoplasy. However, as the posterior region of the other species in the genus has not been fully described, it is not possible to state definitely if it is a valid generic diagnostic character.

According to Ramos (1976), the holotype of *L.nonatoi* was deposited in the collections of the Muséum National d’Histoire Naturelle of Paris (MNHN). However, it is not referred in the MNHN catalogue of polychaete types by Solís-Weiss et al. (2004), neither was it possible to locate it in this museum or in any other collection according to Carrera-Parra (2006b). However, we do not consider it necessary to designate a neotype, as the holotype might still exist in the MNHN collections, being just misplaced or mislabelled. Moreover, this taxon is clearly defined and there is no obvious exceptional need for a neotype designation, in accordance to article 75.1 of the ICZN (International Commission on Zoological Nomenclature 1999).

### ﻿Genus Lumbrineris

Taxon classificationAnimaliaEunicidaLumbrineridae

de Blainville, 1828

A32D666A-0CE4-5F67-B303-722B69168870

#### Type species.

*Lumbrinerislatreilli* Audouin & Milne-Edwards, 1833

#### Diagnosis.

Based on Carrera-Parra (2006a). Prostomium lacking antennae and eyes; notopodia slightly developed, without branchiae. Simple and composite multidentate hooded hooks present. Pygidium with anal cirri. Maxillary apparatus with five pairs of maxillae, carriers as long as MI, joined to entire base of MI; MI forceps-like lacking inner accessory teeth, with attachment lamella; MII as long as MI, with ligament, attachment lamella wide, along 2/3 of posterior lateral edge; wide connecting plates poorly sclerotized; MIII completely pigmented, attachment lamella wide along entire lateral edge; MIV completely pigmented, attachment lamella wide; MV free, reduced to attachment lamella only, lateral to MIII and MIV. Mandibles fused up to 3/4 of their length.

### ﻿ Lumbrineris jan sp. nov.

Taxon classificationAnimaliaEunicidaLumbrineridae

F2554F00-635B-5AAA-B525-8A6A7414340A

https://zoobank.org/07044F02-3757-48A0-97C7-DD9BF7FA3BF1

Fig. 6

Lumbrineris nonatoi [non Ramos, 1976] – Carrera-Parra 2001a: 606, fig. 4A–F; Carrera-Parra 2006b: 45–46, fig. 14F–J; Carrera-Parra 2009: 271.
Lumbrineris sp. D – Uebelacker 1984: 41.44–41.45, figs 41.41–41.42A–H.

#### Type material.

***Holotype***: ECOSUR 298, off Tamaulipas, Mexico, COLT-III E31, 01.05.1992, 34 m. ***Paratypes***: ECOSUR 299, 1 specimen, off Tamaulipas, Mexico, COLT-43 E43, 01.05.1992; ECOSUR 300, 1 specimen, off Tamaulipas, Mexico, COLT-III E19, 01.05.1992; ECOSUR 301, 3 specimens, off North Veracruz, Mexico, 23 m, EMOAPP-II E5, 01.05.1992.

#### Additional material.

As *Lumbrinerisnonatoi*. Gulf of Mexico: **Non-type specimens**: 1 specimen (USNM 75350), off Florida, USA, 26°45'50"N, 82°45'11"W, 24 m, Sofla St. 2B, November 1980; 2 specimens (USNM 90998), off Port Isabel, Texas, USA, 26°10'N, 97°08'W, 15 m, Stocs St. 4/IV-3, Spring 1976; 1 specimen (CP-ICML-UNAM unknown catalogue number), off Veracruz, Mexico, IMCA 2, St. 18, September 1988; 2 specimens (CP-ICML-UNAM unknown catalogue number), off Veracruz, Mexico, IMCA 2, St. 76, 27 September 1988; 2 specimens (ECOSUR-P 1673), off Sontecomapan, Veracruz, Mexico, 18°35'N, 94°52'W, M1E1; 1 specimen (ECOSUR-P 1674), off Punta San Juan, Veracruz, Mexico, 18°20'N, 94°38'W, M1E5; 1 specimen (ECOSUR-P 1675), off Coatzacoalcos, Veracruz, Mexico, 18°11'N, 94°25'W, M1E6; 3 specimens (ECOSUR-P 1676), off Coatzacoalcos, Veracruz, Mexico, 18°20'N, 94°25'W, M1E7; 1 specimen (ECOSUR-P 1677), off Carmen Lagoon, Tabasco, Mexico, 18°23'N, 93°48'W, M1E14; 2 specimens (ECOSUR-P 1678), off Tamiahua Lagoon, Veracruz, Mexico, 21°31'15.78"N, 97°26'59.98"W, 23 m, EMOAPP-II E6, 01.05.1992; 1 specimen (ECOSUR-P 1680), off Tamiahua Lagoon, Veracruz, Mexico, 21°31'59.93"N, 97°28'30.48"W, 52 m, COLT-I E33, 01.05.1992; 1 specimen (ECOSUR-P 1681), Tamaulipas, Mexico, 27 m, COLT-III E11-COSTA, 01.05.1992; 1 specimen (ECOSUR-P 1682), off North Veracruz, Mexico, 56 m, EMOAPP-II E8; 1 specimen (ECOSUR-P 1687), off Campeche, Mexico, 18°58'N, 92°02'W, 20 m, PROMEX-III E30, 14.07.1984; 1 specimen (ECOSUR-P 1688), Campeche, Mexico, 08.08.1984, according to GBIF Secretariat (2021) [probably corresponding to specimen recorded in Carrera-Parra (2001a, 2006b) as “Gulf of Mexico, E7PROMEX-III(1) Gulf of Mexico”]; 1 specimen (ECOSUR-P 1689), off Punta Zapotitlán, Veracruz, Mexico, 18°31'N, 94°36'W, 63 m, PROMEX-III E12, 10.08.1984.

#### Main diagnostic characters.

**Figure 6.**
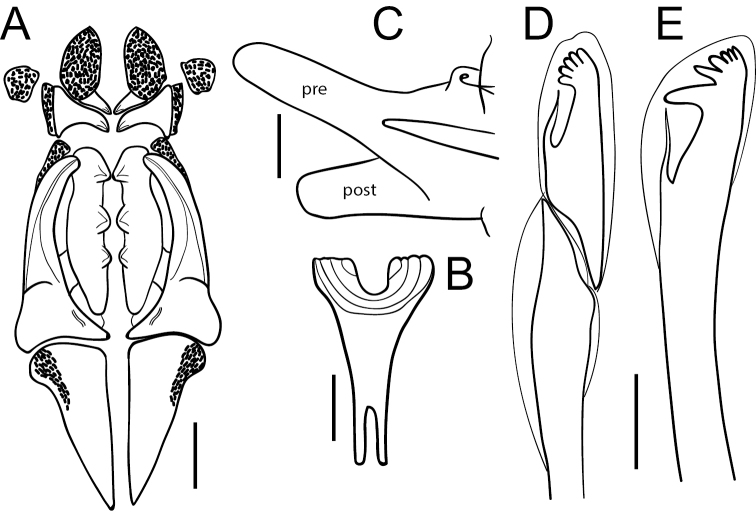
*Lumbrinerisjan* sp. nov. **A** maxillary apparatus **B** mandibles **C** posterior parapodium; pre: prechaetal lobe; post: postchaetal lobe **D** composite hooded hook **E** simple hooded hook **A, C, D, E** redrawn from Carrera-Parra (2006b)**B** redrawn from Carrera-Parra (2001a). Scale bars: 40 µm (**D, E**); 100 µm (**A, B**); 1000 µm (**C**).

Adapted from Carrera-Parra (2001a; 2006b). Prostomium globular, short, ca. as long as wide, lacking antennae and eyes. Peristomium shorter than prostomium, with two rings of similar size. Maxillary apparatus with five pairs of maxillae; maxillary carriers as long as MI, anterior end constricted; MI forceps-like with attachment lamella well developed; MII as long as MI, with wide connecting plates slightly developed and three robust teeth; MIII unidentate; MIV unidentate; MV free, prominent, lateral to MIII and MIV (Fig. 6A). Mandibles translucent, fused for ~ 2/3 of their length (Fig. 6B). Anterior parapodia with prechaetal lobe elongated, gradually increasing to become longer than postchaetal lobe in last body 1/3 (Fig. 6C); postchaetal lobe well developed from first parapodium, digitiform. Composite multidentate hooded hooks up to chaetiger 19, with short blades, having five teeth similar in size and a long hood with a characteristic second swelling at shaft end (Fig. 6D); from chaetiger 20, replaced by simple multidentate hooded hooks, with six teeth, proximal one larger (Fig. 6E). Aciculae yellow, aristate, up to three in anterior parapodia, and one in posterior parapodia. Pygidium with two anal cirri according to Carrera-Parra (2001a), with two rounded lobes and without cirri, according to Uebelacker (1984).

#### Type locality.

Off Tamaulipas, Mexico, Gulf of Mexico (approx. 24°00'N, 97°30'W).

#### Known distribution.

Central Atlantic Ocean, Gulf of Mexico; 15–56 m depth.

#### Etymology.

The specific epithet *jan* is a noun in apposition. The species is dedicated to the loving memory of Jan Ventura Buchaca (2008–2020), a brave young fighter, son of Teresa and Marc and brother of Sara. “*Every time a bell rings an angel gets his wings*”, but Jan got his far too soon.

#### Remarks.

The species was carefully described and illustrated by Carrera-Parra (2001a; 2006b) and thus we are only providing its main diagnostic characters. *Lumbrinerisjan* sp. nov. agrees with *G.nonatoi* comb. nov. in having composite hooks with short blades and a long hood with a characteristic second swelling at shaft end, near the joint with the blade, as well as prechaetal lobes longer than postchaetal ones in most posterior segments. However, it can be clearly distinguished by having a fifth pair of maxillae (four in *G.nonatoi* comb. nov.), blades of composite hooks longer than in *G.nonatoi* comb. nov., five small teeth on composite hooks (vs. 6–8 in *G.nonatoi* comb. nov.) and six small teeth in simple hooks (vs. seven or eight in *G.nonatoi* comb. nov.). Besides, it has two anal cirri (vs. none in *G.nonatoi* comb. nov.), as seen in the description by Carrera-Parra (2001a) and, at least, in the specimen here selected as holotype, and likely corresponding also to the two lobes mentioned and illustrated by Uebelacker (1984).

Carrera-Parra (2001a) highlighted the existence of a size-dependent relationship in the number of chaetigers with composite hooded hooks (i.e., r = 0.864, *P* ≤ 0.01), which agrees with the results of our morphometric analysis based on *G.nonatoi* comb. nov. There are no other morphometric analyses based on the population from the Gulf of Mexico, precluding comparisons. However, it would be very interesting to analyse not only whether differences may exist in the size-related relationships characterizing these two species, but also among other species of *Lumbrineris*.

*Lumbrinerisjan* sp. nov. differs clearly from all previously described species of *Lumbrineris* in having MIII unidentate, aciculae yellow, anterior parapodia with postchaetal lobe digitiform and posterior parapodia with the prechaetal lobe longer than the postchaetal one (Carrera-Parra 2006b; Cai and Li 2011; Carrera-Parra et al. 2011; Martins et al. 2012). All the other known species of *Lumbrineris* with MIII unidentate and aciculae yellow show either or both anterior parapodia with postchaetal lobe auricular and postchaetal lobe always longer than the prechaetal one.

## ﻿Discussion

### ﻿*Lumbrinerisnonatoi* vs. *Gallardonerisnonatoi*

The origin of the misconception leading to the description of *G.iberica* and its successive records across the European Atlanto-Mediterranean waters seems to be clear. It lays mainly in the redescription of “*Lumbrinerisnonatoi*” based on specimens collected from the Gulf of Mexico (Carrera-Parra 2001a, 2006b), which inadvertently introduced a misidentification for the European *L.nonatoi*. A more suitable approach to the supposed lack of type material would have been to rely on the original description of *L.nonatoi* by Ramos (1976), complementing it with new material from the type locality or nearby Mediterranean waters. The misidentified Mexican Gulf specimens used to redescribe “*Lumbrinerisnonatoi*” showed five pairs of maxillae, thus justifying being assigned to *Lumbrineris* (Carrera-Parra 2001a, 2006b), while the specimens used in the original description showed only four (Ramos 1976), allowing it to be placed in *Gallardoneris*.

It should be considered that species descriptions of *Lumbrineris* and other lumbrinerids previous to the 1970s habitually did not report maxillae V. This structure was generally not recognized as a maxilla, but rather referred to as a lateral element or a lateral support, if mentioned at all. This was also the case with Ramos (1976), who counted four pairs of maxillae for all species of *Lumbrineris* she described (as MI, MII, D1, D2), while variously reporting accessory elements. Accordingly, Carrera-Parra (2001a, 2006b) may have assumed that maxillae V were present in the Mediterranean specimens of *L.nonatoi* sensu Ramos (1976), but not mentioned in the description.

The genus *Gallardoneris* was considered as monophyletic by Borisova and Budaeva (2022). However, these authors also stated that the specimens used in their analysis, provisionally identified as *G.iberica* and collected from western African Atlantic waters (i.e., two from Morocco, one from Mauritania and one from Senegal) showed a similar morphology, but clearly diverged molecularly. Accordingly, Borisova and Budaeva (2022) suggested the possible existence of a cryptic complex within the genus that would require further analyses based on a larger number of specimens. Despite we agree with the necessity of further studies, at present this does not contradict the fact that the comparison of the original descriptions of *G.iberica* by Martins et al. (2012) and *L.nonatoi* by Ramos (1976) supports they are the same species. Actually, based on the current knowledge, two aspects sustain the synonymy here proposed. First, the fact that our results are supported by the conclusions of Bertasi et al. (2014) and García Gómez et al. (2016), who compared Mediterranean specimens of *Gallardoneris* with types of *G.iberica* and also concluded that they represented one single species, herein identified as *G.nonatoi* comb. nov. Second, the lack of detailed morphological information on the West African specimens tentatively attributed to *G.iberica* by Borisova and Budaeva (2022) (e.g., there are no data on the presence or number of anal cirri) and the lack of molecular information on the Portuguese and Mediterranean specimens, which does not allow any comparison.

Consequently, the previously reported presence of *Gallardoneris* in European waters (Martins et al. 2012; Bertasi et al. 2014; D’Alessandro et al. 2016; García Gómez et al. 2016; Aguirrezabalaga and Arias 2018; Katsiaras et al. 2018) is here confirmed, but the species representing the genus is, in fact, *G.nonatoi* comb. nov., with *G.iberica* being a junior synonym. Accordingly, the misidentified Mexican Gulf specimens attributed to *L.nonatoi* by Carrera-Parra (2006b) clearly represent a different taxon, which turns out to be a new species that we have named *L.jan* sp. nov.

By clarifying the taxonomic situation of *G.nonatoi* comb. nov., we are also explaining the existence of very numerous Mediterranean reports of *L.nonatoi* previous to the publication of Martins et al. (2012), in contrast to an apparent disappearance of the species in the whole area and its replacement by a proliferation of *G.iberica* (see Bertasi et al. 2014; Katsiaras et al. 2018 and references herein), together with new records of “*Lumbrinerisnonatoi*” in the Gulf of Mexico. By defining the new species and proposing the new combination, we also contribute to solving possible doubts concerning the origin, population fluctuations, and colonization dynamics of the species involved. We are thus showing that the two names represent one single species in European waters, different from the one from the Gulf of Mexico. In light of our observations, their respective distributions remain restricted to their own (original) geographical areas, from where there is no reason to assume they are not native. Therefore, considering the present knowledge, there is no room for discussion of possible introductions or invasions, nor for incorporation of new names to the already growing lists of alien species.

### ﻿On other possible Mediterranean species of *Gallardoneris*

In addition to “*Lumbrinerisnonatoi*”, there is another species deserving to be mentioned in the context of our study. *Lumbrinerislongipodiata* Cantone, 1990 was described from specimens of the Gulf of Catania (Mediterranean Sea), collected in fine sands between 14–16 m depth (Cantone 1990). This is a poorly known species that has been seldom recorded since its short and incomplete (by today’s standards) original description. Though, it could represent an additional member of *Gallardoneris*. The original description reported only four pairs of maxillae, carriers as long as MI, simple and composite multidentate hooded hooks (the latter with short blades), and absence of antennae and branchiae. However, despite some details on the maxillary apparatus were not available, its carriers did not seem to join the entire base of MI and its mandibles were completely fused only along 2/3 of its length, which prevents us to allocate the species in the genus with certainty. Moreover, the identification of further specimens of *L.longipodiata* is eventually hampered by being erroneously keyed with five pairs of maxillae (and so placed within *Lumbrineris*) in the keys available for the region (e.g., Gil 2011; D’Alessandro et al. 2016). A level of confusion still increased by D’Alessandro et al. (2016) further keying out *L.longipodiata* as having bidentate composite hooded hooks, when they are absent in this species. Conversely, it was described as having only four maxillae and thus it should be keyed near to, or even within, *Gallardoneris*. However, many lumbrinerid descriptions lacked information about the presence of MV, which could also be the case with the original description of *L.longipodiata*. Certainly this is critical information that needs to be corroborated.

*Lumbrinerislongipodiata* resembles *G.nonatoi* comb. nov. in having the prechaetal lobe poorly developed and smaller than the postchaetal one in the first chaetigers, of similar size around chaetiger 25 and slightly longer from chaetiger 110, with both lobes becoming cirriform, to twice longer in the last segments. However, it differs in having bidentate MIII, composite hooks to ca. chaetiger 19, and pygidium with two anal cirri (vs. edentate MIII, to ca. chaetiger 9, and anal cirri absent in *G.nonatoi* comb. nov., respectively). Particularly, the differences in mandible shape, fused only in 2/3 of its length in *L.longipodiata* and completely fused in the species of *Gallardoneris* cast additional doubts on its assignment to that genus. The presence of only two anal cirri (also recorded in *L.jan* sp. nov.) is a rare feature among lumbrinerids, being considered as distinctive of *L.longipodiata* (Cantone 1990). Still, despite the number of anal cirri is frequently a size-dependent character (Frame 1992), its single presence (no matter how many) separates clearly *L.longipodiata* from *G.nonatoi* comb. nov., which lacks them. Therefore, in spite of the above-mentioned similarities, the two taxa do not seem to represent the same species. Considering all aspects discussed above, we conclude that it is necessary to redescribe *L.longipodiata* based on topotypic specimens before being able to make a convincing decision on its taxonomic status.

Clearly the knowledge on the European lumbrinerid fauna, especially in the supposedly well-studied Mediterranean Sea, is far from being fully accomplished. Several taxonomic problems remain open, the status of many ancient species has not been revised for decades, and the validity of some of them was never reassessed since their original description or first synonymy, while new names have been introduced for taxa occurring in the same localities. Other records refer to species originally described from distant biogeographic areas and very unlikely occurring in the region. While their records resulted probably from misidentifications derived from the use of bibliography unsuitable to identify the local fauna, they have now entered the local lists of presumed alien and/or introduced species (Langeneck et al. 2020; DM and JG, unpublished data). Definitely, the number of species occurring in the area will keep swinging, especially considering that the acceptance or the rejection of some of them will surely not be consensual among specialists.

## Supplementary Material

XML Treatment for Gallardoneris

XML Treatment for Gallardoneris nonatoi

XML Treatment for Lumbrineris

XML Treatment for Lumbrineris jan

## Supplementary materials

Supplementary material 1 List of character measures used in the morphometric analyses. All measures are expressed in mm except the number of chaetigers and of chaetigers with composite and limbate chaetae Data type Morphometric measurements Explanation note The table includes all morphometric measurements used to analyse the relationships within the examined Mediterranean specimens of *Gallardonerisnonatoi* comb. nov. All data are expressed in mm, except the number of body chaetigers and of chaetigers with composite and limbate chaetae. https://binary.pensoft.net/file/719065This dataset is made available under the Open Database License (http://opendatacommons.org/licenses/odbl/1.0/). The Open Database License (ODbL) is a license agreement intended to allow users to freely share, modify, and use this Dataset while maintaining this same freedom for others, provided that the original source and author(s) are credited.Daniel Martin, Jordi Estefa, João Gil
